# Virtual Reality-Based Therapy Reduces the Disabling Impact of Fibromyalgia Syndrome in Women: Systematic Review with Meta-Analysis of Randomized Controlled Trials

**DOI:** 10.3390/jpm11111167

**Published:** 2021-11-09

**Authors:** Irene Cortés-Pérez, Noelia Zagalaz-Anula, María del Rocío Ibancos-Losada, Francisco Antonio Nieto-Escámez, Esteban Obrero-Gaitán, María Catalina Osuna-Pérez

**Affiliations:** 1 Granada Northeast Health District, Andalusian Health Service, Street San Miguel 2, 18500 Guadix, Spain; icp00011@red.ujaen.es; 2 Department of Health Sciences, University of Jaén, Campus Las Lagunillas s/n, 23071 Jaén, Spain; nzagalaz@ujaen.es (N.Z.-A.); mril0001@red.ujaen.es (M.d.R.I.-L.); mcosuna@ujaen.es (M.C.O.-P.); 3 Department of Psychology, University of Almería, Ctra. Sacramento s/n, 04120 Almería, Spain; pnieto@ual.es; 4 Center for Neuropsychological Assessment and Rehabilitation (CERNEP), Ctra. Sacramento s/n, 04120 Almeria, Spain

**Keywords:** fibromyalgia, virtual reality, physiotherapy, pain, fatigue, quality of life, meta-analysis

## Abstract

Background: Virtual reality-based therapy (VRBT) is a novel therapeutic approach to be used in women with fibromyalgia syndrome (FMS). The aim of our study is to assess the effect of VRBT to reduce the impact of FMS in outcomes such as pain, dynamic balance, aerobic capacity, fatigue, quality of life (QoL), anxiety and depression. Methods: Systematic review with meta-analysis was conducted from a bibliographic search in PubMed, Scopus, PEDro, Web of Science and CINAHL until April 2021 in accordance with PRISMA guidelines. We included randomized controlled trials (RCTs) that compare VRBT versus others to assess the mentioned outcomes in women with FMS. Effect size was calculated with standardized mean difference (SMD) and its 95% confidence interval (95% CI). Results: Eleven RCTs involving 535 women with FMS were included. Using the PEDro scale, the mean methodological quality of the included studies was moderate (6.63 ± 0.51). Our findings showed an effect of VRBT on the impact of FMS (SMD −0.62, 95% CI −0.93 to −0.31); pain (SMD −0.45, 95% CI −0.69 to −0.21); dynamic balance (SMD −0.76, 95% CI −1.12 to −0.39); aerobic capacity (SMD 0.32, 95% CI 0.004 to 0.63); fatigue (SMD −0.58, 95% CI −1.02 to −0.14); QoL (SMD 0.55, 95% CI 0.3 to 0.81); anxiety (SMD −0.47, 95% CI −0.91 to −0.03) and depression (SMD −0.46, 95% CI −0.76 to −0.16). Conclusions: VRBT is an effective therapy that reduces the impact of FMS, pain, fatigue, anxiety and depression and increases dynamic balance, aerobic capacity and quality of life in women with FMS. In addition, VRBT in combination with CTBTE showed a large effect in reducing the impact of FMS and fatigue and increasing QoL in these women.

## 1. Introduction

Fibromyalgia syndrome (FMS) is a chronic disease of unknown etiology that courses with generalized and diffuse non-inflammatory pain and hyperalgesia in different human body points [1]. Other FMS symptoms are joint stiffness [2], generalized fatigue [3], impaired balance [4], anxiety and depression [5], and emotional overload [6] that reduces functional capacity [7] personal autonomy, social relationships [8] and quality of life (QoL) [9,10]. The global prevalence of FMS ranges between 2% and 8% of the population [11], mainly affects women (61–90%) [12] aged 50 years and over [13] and involve a large consumption of social and health resources [14]. The negative impact of FMS has turned FMS into a public health problem that requires the search for a therapeutic approach to reduce the negative impact of FMS symptoms [15].

Although the pathogenesis of FMS is unknown, the mechanisms responsible for dysfunctional pain in FMS, without having identifiable tissue lesions, are mainly related to disorders of the central nervous system (CNS) that may explain diffuse musculoskeletal pain [16]. Several studies have reported numerous changes in the brain cortex and spinal cord descending tracts (i.e., descending pain modulatory system) in patients with FMS producing central sensitization (CS) [17] and deficits in pain inhibitory mechanisms [18]. Several studies have shown that patients with FMS present hyperactivity and hyper-excitability of their CNS, suggesting that it is supported by continuous nociceptive peripheral inputs [19]. Recent neuroimaging and biochemical studies have shown a reduction in serotonin (5HT) and noradrenalin (NA) in cerebrospinal fluid [20] that may support continuous and widespread pain in FMS due to dysfunction in the descending inhibitory systems [21]. In addition, the activity of the insula lobe was shown to increase, producing higher levels of the neurotransmitter glutamate in the posterior insula, which has been associated with chronic pain [22]. Studies have reported evidence regarding peripheral neurogenic inflammation in patients with FMS, compared to healthy subjects, due to the presence of different proinflammatory peptides from the nerve terminals of peptidergic C-fibers, including substance P, calcitonin gene-related protein and neurokinin A [22], all of which are related to vasodilatation and an increase in vascular permeability responsible for maintaining pain in FMS [23].

In recent years, numerous therapeutic proposals have been implemented in an attempt to reduce the symptoms and the impact of FMS [14]. In addition to pharmacotherapy, conservative non-pharmacological interventions based on physiotherapy, conventional therapy (CT), or physical exercise are another modality in the treatment of FMS [24]. Recent studies have highlighted active physical training as an effective therapy to improve balance [25], pain [26], muscle fatigue [27], anxiety [28] and QoL [29], among others, in patients with FMS. However, with the aim to get an increasing in the effect of CT, new technologies have been used. For example, virtual reality-based therapy (VRBT) has experienced growth as a method for physical and cognitive training, showing benefits in different contexts [30]. Virtual reality (VR) technology enables patients to be included in a virtual environment similar to the real world through a computer and interact with it [31]. Immersive VR (iVR) uses headsets to display 3D digital images at 360° that simulate any scenario with high realism, allowing patients to interact with this virtual environment using a hand controller or their own hands [32]. Non-immersive VR (niVR) is considered more accessible and inexpensive than iVR and enables patients to visualize virtual environments in 2D projected onto a screen and interact with them through the use of a mouse, keyboard or joysticks [33,34]. VRBT is considered a useful intervention along with CT for neurological [35] or musculoskeletal disorders [36] that can be used at home, thereby favoring patient accessibility to physiotherapy protocols (telephysiotherapy), which has been especially important during the COVID-19 pandemic [37].

The usability and feasibility of VRBT in CT protocols with promising results in the management of pain, anxiety or mood states have been shown [38]. For example, in patients with FMS, VRBT has allowed specific, intensive, multisensory and active therapies with quick feedback in different environments and situations to be performed that increased the motivation of the patient [39] and adherence to the therapy [40]. However, the absence of a systematic review (SR) that unifies the available knowledge on the use and effects of VRBT in patients with FMS may limit the impact of VRBT use. Therefore, the aim of our research is to assess the effect of virtual reality-based therapy (VRBT) reduce the disabling impact of fibromyalgia syndrome (FMS). In addition, to know if the effect shown by VRBT is greater when VRBT is used alone or in combination with other therapies on FMS.

## 2. Materials and Methods

### 2.1. Protocol Design

This systematic review is reported in accordance with the preferred reporting items for systematic reviews and meta-analyses (PRISMA) statement [41] and it was, previously registered in PROSPERO: CRD42021225635.

### 2.2. Search Strategy and Data Sources

Two authors (I.C.-P. and M.C.O.-P.), independently, performed a bibliographical search in PubMed Medline, Web of Science (WOS), Scopus, CINAHL Complete and PEDro (Physiotherapy Evidence Database) to select articles published up to April 2021. The authors reviewed the reference lists from retrieved full-text studies and previously published grey literature, expert documents and congress abstracts. Based on Medical Subjects Headings (MeSH) the keywords used in the search strategy were “fibromyalgia”, “virtual reality” and “virtual reality exposure therapy” with its synonyms. Based on the particular database, the Boolean operators “AND”/“OR” were used to combine the keywords and entry terms, according to PICOS tool of Cochrane Collaboration [42], for the retrieval of reports of randomized controlled trials (RCTs). Language and publication date filters were not used. A third expertise author (E.O.-G.) revised the bibliographic search and resolved doubts. Table 1 shows the search strategy used in each database. 

### 2.3. Study Selection and Inclusion Criteria

Two blinded reviewers (N.Z.-A. and F.A.N.-E.), independently, screened the titles and abstracts of all references retrieved in each database. When one of these authors selected an article during the inclusion phase based on the title and abstract, it was examined in detail. All disagreements were resolved by a third author (M.C.O.-P.). A study was included in the present SR when it met all the following inclusion criteria: (1) RCTS or RCT pilot study; (2) comprised by women with FMS; (3) with at least two groups; (4) of which one group received VRBT; (5) compared with controls; (6) and reported quantitative data of different outcomes related with FMS impact (see Section 2.5). The exclusion criteria that were established included (1) experimental studies without a comparison group and (2) studies comprising patients with a variety of musculoskeletal disorders (i.e., not only FMS).

### 2.4. Data Extraction

Two authors (I.C.-P. and E.O.-G.) independently collected data from the included studies in a standardized Microsoft Excel data-collection form. To resolve disagreements, a third author was consulted (M.C.O.-P.). We extracted the following data: (1) overall characteristics of the study (authorship and publication date, study design, number of groups, total sample size and time since FMS diagnosis); (2) characteristics of the intervention and control groups (number of participants, mean age, sex, body mass index, intervention and duration of the intervention in weeks, number of sessions per week and duration of each session in minutes); (3) data related to the post-intervention outcomes (outcomes assessed, mean and SD of each outcome in each study and group); and (4) evaluation time sequence (right at the end of the therapy or follow-period). When a study did not provide statistics appropriate for performing the meta-analysis, we extracted the median, standard error, range or interquartile range to be transformed into a SD [42]. 

### 2.5. Outcomes

The main outcome assessed in this SR was the impact of FMS, which was assessed in patients with FMS after receiving VRBT in comparison to other interventions (such as conventional therapy-based therapeutic exercise (CTBTE) or stretching [ST]) or no intervention (NI). The Fibromyalgia Impact Questionnaire (FIQ) was selected to assess the impact of FMS in the selected studies. In addition, other variables assessed in this SR were pain, dynamic balance, aerobic capacity, fatigue, QoL, anxiety and depression. Different assessments examining the same outcome were grouped together for analysis (see results of meta-analyses section).

### 2.6. Risk of Bias and Methodological Quality Assessment

The PEDro Scale was used to assess the risk of bias of the included studies [43]. The PEDro Scale comprised 11 items with two answer options (“yes” if the criterion was clearly satisfied and “no” if the criterion was not satisfied) [44]. The total score could vary across a range from 0 (high risk of bias) to 10 (low risk of bias), and item 1 was not used for its relationship with external validity [45]. 

The Grading of Recommendations Assessment, Development, and Evaluation (GRADE) assessment [46] was used to analyze the quality evidence of the findings. This scale assesses the risk of bias in each study, inconsistency, indirectness, precision and the risk of publication bias. All these items, except the risk of bias, were assessed using the GRADE checklist of Meader et al. (2014) [47] Inconsistency was assessed based on heterogeneity level [48]; precision was assessed through the number of participants per study (large > 300 participants, moderate 300–100 participants and low < 100 participants) and with the number of studies included (large >10 studies, moderate 10–5 studies and low < 5 studies) [46]; and indirect evidence was considered to exist in those articles in which the results were indirectly measured and was scored as “yes” or “no”. Risk of publication bias is explained in statistical analysis Section 2.7.

Two authors (N.Z.-A. and M.d.R.I.-L.) independently assessed the risk of bias, and quality evidence and doubts related to this assessment were resolved in consultation with a third researcher (M.C.O.-P.). The quality evidence of each meta-analysis was downgraded from high quality by one level for each factor that was found. In the case of the presence of several limitations, the overall quality level was downgraded by two levels. Finally, the level of evidence in each meta-analysis was categorized as (1) high: the findings are robust; (2) moderate: it is possible that new research may change our results; (3) low; the level of confidence in our pooled effect is very slight; or (4) very low: any estimate of effect is very uncertain.

### 2.7. Statistical Analysis

Two authors (E.O.-G. and I.C.-P.) carried out the meta-analysis using Comprehensive Meta-Analysis version 3.0 (Biostat, Englewood, NJ, USA) [49]. Meta-analysis was conducted only if an outcome measure was provided by at least two studies. To perform the meta-analysis, we followed the recommendations of Cooper et al. (2009) [50] and to estimate the effect of VRBT, Cohen’s standardized mean difference (SMD) [51] and its 95% confidence interval (95% CI) in a random effects model proposed by DerSimonian and Laird [52] were used. Cohen’s SMD can be interpreted as one of four effect strength levels: no effect (SMD 0), small (SMD 0.2–0.4), medium (SMD 0.4–0.7) and large (SMD > 0.8) [53]. In addition, when an outcome was assessed with the same test, we calculated the mean difference (MD) with the aim of comparing our results to the minimal clinically important difference (MCID) value for each assessed outcome. The pooled effect of each meta-analysis is displayed using forest plots [54]. The risk of publication bias was assessed through the visualization of symmetric (low risk) or asymmetric (high risk) funnel plots [55] using Egger’s test (where if the *p*-value < 0.1, there exists a risk of publication bias) [56]. In addition, the trim-and-fill method was used to estimate the adjusted SMD, taking into account any possible risk of publication bias [57]. Based on Rothman’s recommendations for the effect size variation limit in the assessment of confusion bias, when the adjusted SMD varied more than 10% with respect to the original and raw pooled effects, the quality level of evidence was downgraded one level, although the funnel plot was slightly asymmetrical [58]. Finally, the level of heterogeneity was assessed with the *Q*-test and the degree of inconsistency (I^2^) from Higgins et al. [48]. Heterogeneity may be present when the *p*-value < 0.1, and it can be categorized as low (I^2^ < 25%), moderate (I^2^ between 25–50%) or large (I^2^ > 50%) [59,60].

### 2.8. Additional Analyses

To assess the effect of the use of VRBT alone or combined with CTBTE on the impact of FMS, we performed a subgroup analysis (VRBT + CTBTE vs. CTBTE or VRBT vs. NI) using data from RCTs. In addition, a sensitivity analysis was performed using the leave-one-out method [42,50] that shows how each individual study affected the overall estimate based on the remaining studies. Finally, a qualitative synthesis was carried out for those variables that did not report data that could be meta-analyzed but were analyzed in the studies included in the review.

## 3. Results

### 3.1. Study Selection

**Figure 1:**
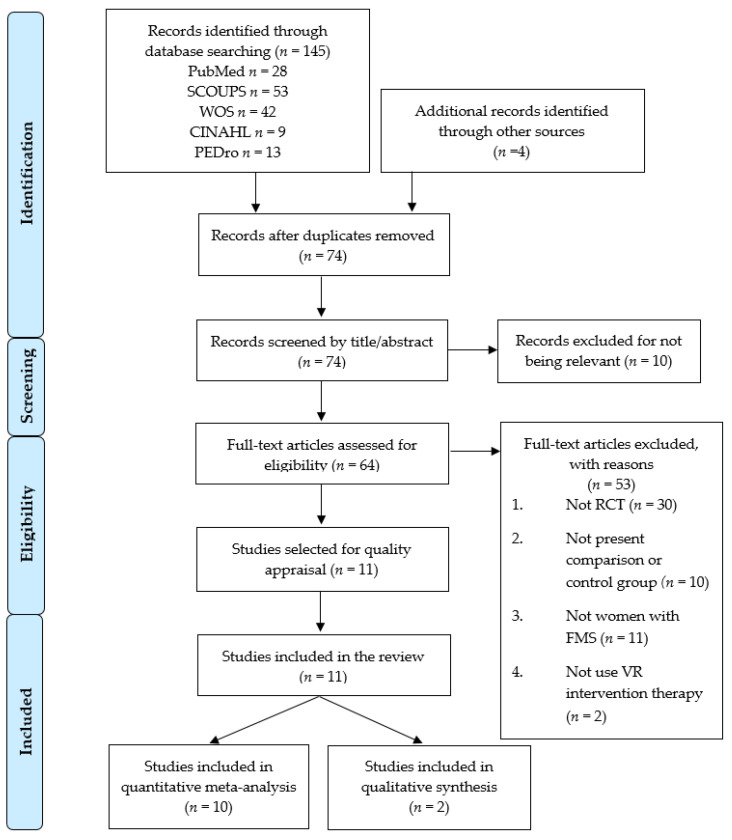
Preferred reporting items for systematic reviews and meta-analyses (PRISMA) flow chart for the systematic literature search and study selection process. Note: One study (León-Llamas, JL et al. 2020) [67] provided information for quantitative and qualitative synthesis.

The PRISMA flow chart (Figure 1) displays the study selection process. One hundred forty-nine records were retrieved from the initial bibliographical search (145 from databases and 4 in others additional sources). Seventy-five studies were excluded for duplication, and 74 references were initially screened by title and abstract. Ten studies were removed based on title/abstract, and 53 studies did not meet the inclusion criteria. Finally, 11 RCTs [61,62,63,64,65,66,67,68,69,70,71] were included. Specifically, 10 RCTs [61,62,64,65,66,67,68,69,70,71] were included in the quantitative synthesis, and 2 RCTs [63,67] were included in the qualitative synthesis. One RCT [67] provided information for quantitative and qualitative synthesis.

### 3.2. Characteristics of the Studies Included in the Review

All included RCTs were carried out between 2015 and 2021 (2015 [65], 2017 [62,64], 2019 [63,68,70,71], 2020 [61,66,67] and 2021 [69]) in Spain [62,63,64,65,67,68,70,71], Turkey [66,69] and Brazil [61]. The included studies reported data from 535 participants with FMS (100% women) with a mean age of 51.11 ± 4.2 years old, a mean BMI of 27.27 ± 1.6 kg/m^2^ and a mean duration of FMS symptoms of 10.49 ± 5.4 years. Two hundred seventy-nine women (51.68 ± 3.9 years old) were in the experimental intervention groups receiving VRBT, and 256 participants (50.54 ± 4.6 years old) were in the control intervention groups. In the experimental group, 10 RCTs used non-immersive VRBT [61,62,63,64,65,67,68,69,70,71] and 1 RCT [66] used immersive VRBT. In addition, VRBT was used alone in 9 RCTs [61,62,63,64,65,67,68,70,71] and in combination with CTBTE in another 2 RCTs [66,69]. Regarding the control groups, in 2 RCTs, women received CTBTE (aerobic exercise [69] and Pilates therapy [66]); in one RCT [61], women received ST; and in 8 RCTs [62,63,64,65,67,68,70,71] participants did not receive any therapy. The duration of VRBT in weeks was heterogeneous at 3 [65], 7 [61], 8 [62,64,66,69] and 24 weeks [63,67,68,70,71]; the number of sessions per week was 2 [62,63,64,65,66,67,68,70,71] and 3 [61,69] and finally, the duration of each session in minutes was 60 min [61,62,64,65,67,68,70,71], 35 min [69] or 80 min [66]. All assessments were carried out to the end of the intervention. Table 2 shows the characteristics of the studies included in this SR.

### 3.3. Risk of Bias Assessment of the Studies Included in the Review

The risk of bias scores for 7 RCTs [61,62,63,64,68,70,71] were obtained on the PEDro website, and for four RCTs [65,66,67,69] it was assessed manually. All included RCTs obtained a score of at least 5 points. The mean PEDro score was 6.63 ± 0.51 points. The impossibility of blinding the participants and the therapist and participants’ concealed allocation were the items involved in the high risk of bias. Table 3 shows the PEDro assessment.

### 3.4. Quantitative Synthesis

Ten RCTs [61,62,64,65,66,67,68,69,70,71] with 34 independent comparisons providing data for 485 women with FMS (50.92 ± 4.4 years old, mean BMI of 27.27 ± 1.59 kg/m^2^ and a mean duration of symptoms of 10.49 years) were included in the quantitative synthesis. Table 4 shows the main findings of each meta-analysis.

#### 3.4.1. Impact of FMS Symptoms

**Figure 2:**
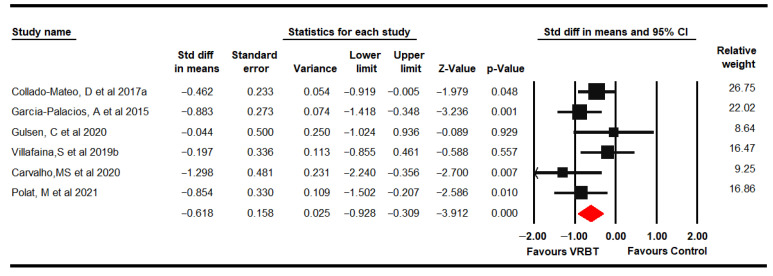
Forest plot of the effect of virtual reality-based therapy from the Fibromyalgia Impact Questionnaire.

Six RCTs [61,62,65,66,69,71] provided data from 249 women with FMS (49.3 ± 5.06 years old), in which the impact of FMS was assessed using the FIQ-total score. Moderate-quality evidence suggested a medium effect of VRBT on FIQ scores (SMD −0.62, 95% CI −0.93 to −0.31; *p* < 0.001) (Figure 2) showing a reduction of FIQ of −9.96 (95% CI −13.64 to −6.28) compared to control, favored VRBT. No risk of publication bias (Egger *p* = 0.9) or heterogeneity (I^2^ 5.18%) were found. Sensitivity analysis did not reported variation.

Subgroup analysis showed a reduction in FIQ scores favors VRBT (MD −9.01, 95% CI −14.13 to −3.88) compared to NI and in the combined use of VRBT + CTBTE (MD −9.86, 95% CI −17.65 to −2.06) in comparison to CTBTE, with low- and very low-quality evidence, respectively.

#### 3.4.2. Pain

**Figure 3:**
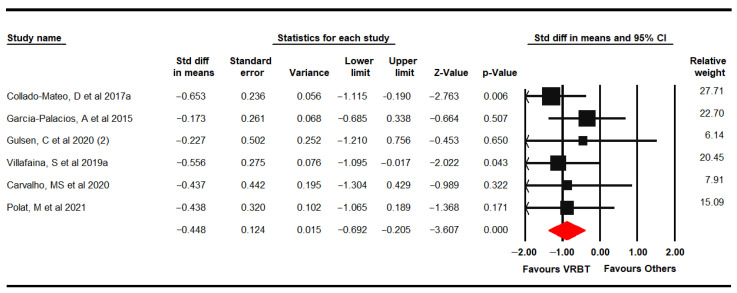
Forest plot of the effect of virtual reality-based therapy on pain.

Six RCTs [61,62,65,66,69,70] provided data on 267 women with FMS (49.28 ± 5.04 years old) using the VAS, the pain dimension on the FIQ and the Brief Pain Inventory (BPI). Low-quality evidence showed a moderate effect of VRBT (SMD −0.45, 95% CI −0.69 to −0.21; *p* < 0.001) compared to controls (Figure 3). The risk of publication bias may be considered (Egger *p* = 0.2 and trim-and-fill variation of 28%) without heterogeneity (I^2^ 0%). Sensitivity analysis showed a variation of 17% when Collado-Mateo [62] was removed.

Subgroup analysis showed low-quality evidence of a medium effect of VRBT in comparison with NI (SMD −0.64, 95% CI −1.18 to −0.11), favored VRBT.

#### 3.4.3. Dynamic Balance

**Figure 4:**
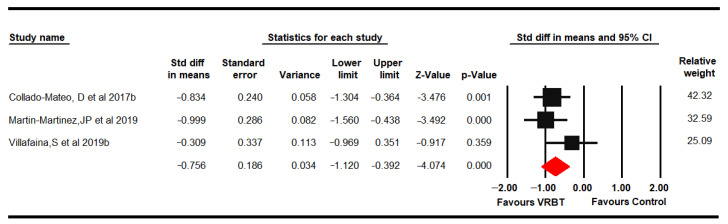
Forest plot of the effect of virtual reality-based therapy on dynamic balance.

Three RCTs [64,68,71] reported data from 168 women with FMS (53.36 ± 0.74 years old), which assessed dynamic balance using the Timed Get Up and Go Test (TGUGT) and the 10-Step Chair Test (SCT). Low-quality evidence of a medium-high effect of VRBT (SMD −0.76, 95% CI –1.12 to −0.39; *p* < 0.001) on dynamic balance in comparison with NI was found (Figure 4). No risk of publication bias (Egger *p* = 0.52) and low heterogeneity were present (I^2^ 4.35%). There was an estimated variation of 20% with respect to the original SMD when Villafaina was excluded [71]. In this outcome, subgroup analysis was not performed due to lack of studies for other comparisons.

#### 3.4.4. Aerobic Capacity

**Figure 5:**
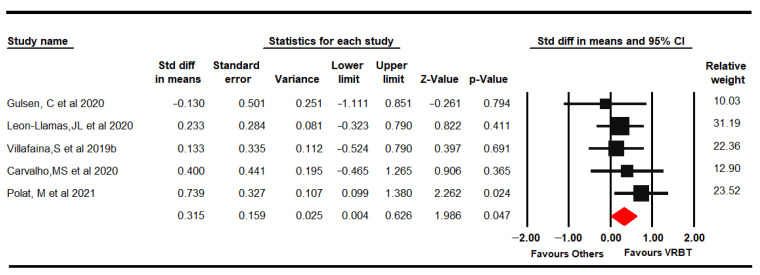
Forest plot of the effect of virtual reality-based therapy on aerobic capacity.

Five RCTs [61,66,67,69,71] provided data from 164 women with FMS (49.26 ± 5.72 years old) to assess aerobic capacity with the 6-minute walk test (6-MWT) and oxygen volume partial pressure (PVO_2_). Low-quality evidence of a small effect of VRBT (SMD 0.32, 95% CI 0.004 to 0.63; *p* = 0.047) on aerobic capacity was shown compared to other therapies or NI (Figure 5) without heterogeneity (I^2^ 0%). The risk of publication bias was low (Egger *p* = 0.31 and trim-and-fill variation of 12%). Sensitivity analysis showed a variation of 41% with respect to the original pooled effect when excluding Polat [69].

Subgroup analysis was performed for the comparison VRBT vs. NI, without find statistical significant between therapies (SMD 0.18; 95% CI −0.17 to 0.54; *p* = 0.31). 

#### 3.4.5. Fatigue

**Figure 6:**
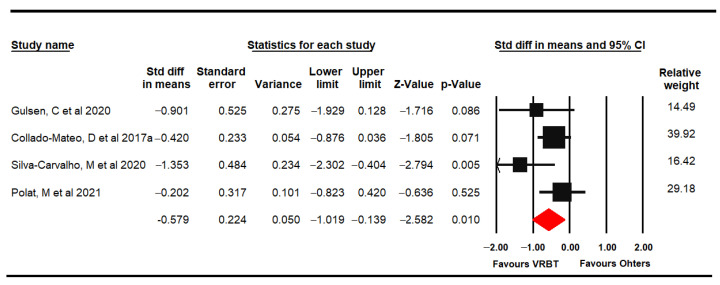
Forest plot of the effect of virtual reality-based therapy on fatigue.

Four RCTs [61,62,66,69] reported data from 153 women with FMS (47.86 ± 5.62 years old) that assessed fatigue through the FIQ fatigue domain and Fatigue Severity Scale (FSS). Low-quality evidence showed a medium effect of VRBT (SMD −0.58, 95% CI −1.02 to −0.14; *p* = 0.01) on fatigue compared with other interventions or NI (Figure 6) with low heterogeneity (I^2^ 5.4%). Risk of publication bias was present (Egger *p* = 0.09 and variation of 20% with the trim-and-fill method). Sensitivity analysis showed a variation of 31% when excluding Polat [69].

Subgroup analysis showed low-quality evidence of a reduction in fatigue (MD −2.21 95% CI −4.33 to −0.1) when VRBT was used in combination with CTBTE compared to CTBTE alone, favored VRBT intervention.

#### 3.4.6. Quality of Life

**Figure 7:**
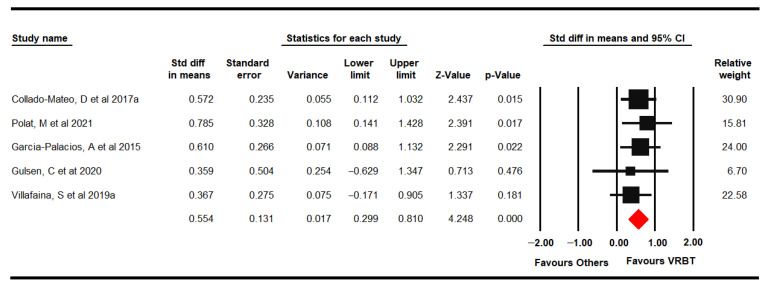
Forest plot of the effect of virtual reality-based therapy on quality of life.

Five RCTs [62,65,66,69,70] provided data from 246 women with FMS (48.8 ± 5.1 years old) that assessed QoL with the EuroQol-5D, QoL Index and SF-36. Moderate-quality evidence of a medium effect of VRBT (SMD 0.55, 95% CI 0.3 to 0.81; *p* < 0.001) on QoL was shown in comparison to CTBTE or NI (Figure 7). No risk of publication bias or heterogeneity was found (I^2^ 0%). Sensitivity analysis did not show variation.

Subgroup analysis showed a medium effect (SMD 0.48, 95% CI 0.19 to 0.78) of VRBT and VRBT + CTBTE (SMD 0.66, 95% CI 0.12 to 1.2) compared to NI and CTBTE, respectively, with low-quality evidence.

#### 3.4.7. Anxiety and Depression

**Figure 8:**
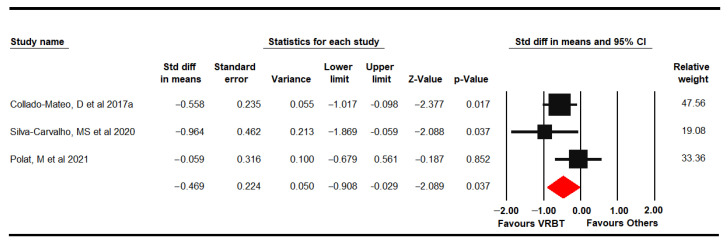
Forest plot of the effect of virtual reality-based therapy on anxiety.

Anxiety was assessed in 3 RCTs [61,62,69] that provided data from 137 women with FMS (49.65 ± 4.74 years old) using the FIQ anxiety domain and Hospital Anxiety and Depression Scale, anxiety dimension (HADS-A). Very low-quality evidence of a medium effect of VRBT (SMD −0.47, 95% CI −0.91 to −0.03; *p* = 0.037) in comparison to other interventions or NI (Figure 8) without heterogeneity (I^2^ 0%) and with a possible risk of publication bias (trim-and-fill variation of 22%). Sensitivity analysis showed a variation of 36% when excluding Polat [69]. In this outcome, subgroup analysis was not performed due to lack of studies for other comparisons (only one study was included per specific comparison).

**Figure 9:**
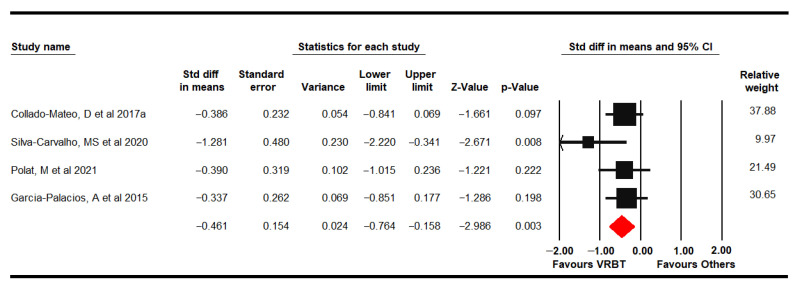
Forest plot of the effect of virtual reality-based therapy on depression.

Four RCTs [61,62,65,69] provided data from 196 women with FMS (49.86 ± 4.02 years old) in which depression was assessed using the FIQ depression domain, HADS depression dimension and Beck Depression Inventory (BDI-II). Low-quality evidence of a medium effect of VRBT (SMD −0.46, 95% CI −0.76 to −0.16; *p* = 0.003) was shown compared to other interventions or NI (Figure 9). A possible risk of publication bias would be considered due to a variation of 13% with the trim-and-fill method (Egger *p* = 0.14) and low heterogeneity (I^2^ 4.59%). Sensitivity analysis displayed a variation of 19% with respect to the original pooled effect when Silva-Carvalho was excluded [61]. A subgroup analysis could only be performed in the comparison VRBT vs. NI, showing a medium effect (SMD −0.56; 95% CI −0.97 to 0.15; *p* = 0.008). 

### 3.5. Qualitative Synthesis

Two RCTs [63,67] assessed changes in brain structures but did not report data appropriate for performing a meta-analysis, so a qualitative synthesis was made. León-Llamas, J.L. et al., 2020 [67] evaluated the effects of VRBT on grey matter volume in different brain structures in 50 women with FMS through magnetic resonance imaging (MRI). Significant relationships (*p* < 0.05) between PVO2 and the left and right regions of the hippocampus and the left and right regions of the amygdala were found with VRBT compared to NI. Villafaina, S. et al., 2019a [63] evaluated the effects of VRBT on resting brain dynamics in 55 women with FMS. Significant differences in measured electroencephalographic (EEG) signals were found in some frontal, parietal, temporal and occipital areas (*p* < 0.05) in the VRBT group. VRBT was more effective in the group with a shorter duration of symptoms, showing between-group differences in some frontal and temporal areas.

## 4. Discussion

FMS is a chronic disease characterized by widespread muscle pain that reduces functional capability and QoL. In addition to other therapies, such as pharmacotherapy [72], balneotherapy [73] or physical exercise [74,75], VRBT has emerged as a new therapy that can reduce the impact of FMS symptoms. The present study proposed to compile the scientific evidence published to date to analyze the effectiveness of VRBT on the impact of FMS and in other related outcomes.

One of the most important impairments of FMS is its impact on ADLs, which is mainly assessed with the FIQ [76]. Our results suggested that VRBT is effective to reduce the impact of FMS symptoms on ADLs. The significant decrease in FIQ scores (approximately 10% of the total score both with VRBT alone or combined with CTBTE) would be related to the fact that VRBT is an active therapy that requires continuous body movements, which can combine the effect of multisensory stimulation and physical exercise. VRBT is considered a positive, different and motivating experience that directly impacts well-being and reduces the perception of the severity of symptoms. These results support the fact that VRBT may be considered a real therapeutic option to reduce the impact of FMS. In addition, subgroup analysis according to specific comparisons, showed that to VRBT is better than or NI (also called, UC) and the combined use of VRBRT with CTBTE is better that to perform only CTBTE. Between these two comparisons, VRBT with CTBTE is the best therapeutic option to improve the FIQ in women with FMS. It can allow personalize therapies combining different VR videogames and CTBTE protocols adapted for each women with FMS.

Some studies have suggested that VRBT is a novel approach in the field of orthopedic rehabilitation [77], in the management of acute or chronic pain [78] and in neuropathic pain control [79], among others. Our findings showed that VRBT could be effective as a non-pharmacological alternative intervention to reduce pain in women with FMS. Subgroup analysis revealed that in comparison with UC, VRBT is effective to reduces the pain level. The high levels of body pain that patients with FMS suffer from are supported by CS, which can be enhanced or maintained by supraspinal processes involving cognitions and focused attention on the sensation of pain [80]. It has been described that emotions that are directly connected to the limbic system can modulate pain through descending pathways [81]. To interact with VRBT as a method of distraction in which many neurophysiological connections occur between the visual and somatosensory systems might divert attention, leading to a slower response to incoming pain signals [78,81]. Likewise, a pleasant playful experience would lead to positive emotions capable of improving endogenous nociceptive inhibition. The continuous pain experience in patients with FMS produces muscle debility that together with negative emotions when moving can favor the appearance of kinesiophobia that reduces active movement and muscle tone, thereby increasing fatigue and pain [82]. VRBT requires autonomous movement of different body parts that can increase muscle tone and the perception of pain-free movement favoring the elimination of restrictions to movement included in the cerebral body scheme due to continued pain. Performing CTBTE through VR devices could provide greater adherence by using exercises adapted to different levels of progression. VR-based exercises could be more enjoyable and stimulating, facilitating implementation in subjects with FMS who have difficulty adhering to CTBTE.

Balance disorders appear in women with FMS, increasing the risk of falls [4]. A recent meta-analysis showed that women with FMS develop balance disorders [83] resulting in a high risk of falls. Some studies have found a reduction in brain grey matter in which vestibular, visual and somatosensory information is processed and integrated to produce a balanced response [84]. For this, it is necessary to implement therapies that provide the patient with multisensory stimulation at the same time that the patients are forced to actively work. Our results suggested that VRBT improves dynamic balance in subjects with FMS. VRBT is an active therapy that requires engaging in physical activities during the sessions, and the findings obtained were similar to those in different reviews that assessed the effects of physical exercise or Tai Chi to increase the balance in FMS women [25,85]. According to Villafaina, S. et al., 2019 [63], VRBT produces changes in different brain areas and increases the grey matter and EEG signals in the frontal, parietal, temporal and occipital lobes, some of which are responsible for integrating the balance information needed to respond to a destabilizing stimulus or Earth’s gravitational force. The continued and active work in the standing position with a high level of multisensory stimulation by VR devices improve the neuromuscular efferent responses to maintain balance. Finally, some patients with FMS take anxiety and depression drugs, such as antipsychotics, that can increase the risk of falls [86]. VRBT includes virtual physical exercise to improve muscle tone, postural afferences and neuromotor responses to an antigravitational stimulus, reducing the risk of falls.

Women with FMS reported a decreased aerobic capacity, which could have a negative impact on functional capacity and consequently on QoL. Our findings showed an improvement in aerobic capacity in women who were exposed to VRBT. VRBT could provide the training of representative tasks included in ADLs that require less muscle strength and allow individuals to perform the activity for longer periods of time, which increases the cardiorespiratory fitness of these patients [7].

Generalized fatigue induces a sedentary lifestyle in women with FMS, which reduces aerobic capacity [87]. For this reason, women with FMS can perceive higher levels of fatigue during the performance of ADLs. Our results suggested that VRBT is useful for reducing fatigue in women with FMS, and there was a greater effect when combined with exercise. Our results are in line with a recent review in which VRBT has been postulated to be an effective method to reduce fatigue in different groups of patients [88]. In recent years, numerous therapies have emerged for the treatment of fatigue in FMS; however, the provided evidence has not been sufficient to unify criteria regarding the most appropriate treatment [89]. In this sense, VRBT may be an effective therapy to reduce fatigue because it is a customized method that increases distraction and prevents patients from being aware of fatigue.

In our study, we report that QoL improves after to use of VRBT. Restoring and balancing physical activity is a challenge in patients with FMS and chronic pain. Low mood, pain, and fatigue decrease the willingness to perform ADLs, causing low motivation and a poor sense of self-efficacy and QoL. Recent studies have shown that VRBT improves mood states, positive emotions, motivation, and self-efficacy in FMS patients [39], which should have a positive impact on QoL. A recent review postulated that the regulation of emotions through VRBT could be an effective method to increase QoL and personal well-being [90]. In addition, several studies have analyzed the efficacy of VRBT in combination with other therapies on QoL in different pathologies, such as rheumatic and orthopedic diseases, finding beneficial effects [77].

Our results showed that the levels of anxiety and depression improved after the use of VRBT in women with FMS, although a major effect was found on depression outcomes. Physical exercise in FMS patients has previously been shown to improve anxiety [91] and depression [92]. In recent years, VRBT has been considered a new and cost-effective tool to complement psychological treatments [93]. Our findings are consistent with a recent meta-analysis that assessed the effect of VRBT on anxiety and depression, finding a reduction in anxiety and depression after the application of VRBT in comparison to control conditions (e.g., waitlist, placebo, relaxation or NI) [94].

One study included in our review found significant relationships between pVO_2_ and the left and right regions of the hippocampus and the left and right regions of the amygdala after training with VRBT [67]. These findings may be in line with the results of a study that analyzed neural activity and respiratory frequency in anticipation of anxiety. The activation of this area participates in the enhancement of respiratory frequency. Electric current sources were found in the left amygdala in the most anxious subjects [95]. The authors pointed out the need to study the relationship between the aerobic system and the amygdala, since women with FMS constitute a population associated with anxiety symptoms [96,97]. Findings from Villafaina, S et al., 2019 [63] indicated that VRBT can produce changes in the dynamics of the brain that could be related to an increase in cerebral blood flow. These results are particularly relevant to FMS patients because they frequently have altered cerebral blood flow variability and velocity [98,99], as well as impaired cognitive function [100]. Thus, VRBT in FMS patients could increase cerebral blood flow and, consequently, cognitive function. Additionally, the intervention in this study was more effective in the group with a shorter duration of symptoms, showing between-group differences in some frontal and temporal areas. Previous studies have shown that a longer duration of FMS symptoms predicts lower FIQ scores, which shows that patients who have suffered from FMS symptoms for short periods of time may be more severely affected by the disease [101]. These results suggested that VRBT could counteract severe FMS symptoms.

Our results may be considered to have some limitations. First, the low number of studies included may make generalization of our findings difficult. Related with the generalization of our findings a limitation is that our findings are applied to women with an age that varied between 38 and 55 years old (mean of 51.4 years old) and BMI between 25 to 28 (mean of 27.7). It would be important to perform new studies in all groups of age. Second, the impossibility of blinding the therapy to participants increased the risk of selection bias. Third, it is important to consider the possible risk of publication bias that may have reduced the reported effects of therapy. Another limitation was the low number of participants per study, which reduced the precision level of our findings. Besides, it is important to highlight that the large variations in sensitivity analysis may have affected the quality of our findings. Finally, it is important to remark that all studies were performed in Spain, Turkey or Brazil and it can affect to the generalization of our findings. It would be necessary to perform studies in more countries to increase the application of our findings.

## 5. Conclusions

This is the first SR with meta-analysis that demonstrates the effect of VRBT in women with FMS in reducing the health impact of symptoms of FMS, in short-term follow up. Our results showed an effect of VRBT in comparison to other interventions or NI and favored VRBT intervention based on the impact of FMS, pain, dynamic balance, aerobic capacity, fatigue, QoL, anxiety and depression. In addition, when VRBT was combined with CTBTE and compared to CTBTE alone, our findings showed a large effect of VRBT + CTBTE intervention on the measures of the impact of FMS fatigue and QoL. However, it is necessary to conduct further research on this topic, while increasing the sample size and extending the assessments to the long term.

## Figures and Tables

**Table 1:** Search strategy used in each database.

Databases	Search Strategy
PubMed Medline	(fibromyalgia[mh] OR fibromyalgia[tiab] OR fibromyalgia syndrome[tiab] OR fibromyalgia*[tiab] OR chronic, fatigue syndrome[tiab]) AND (virtual reality[mh] OR virtual reality[tiab] OR virtual reality exposure therapy[mh] OR virtual reality exposure therapy[tiab] OR exergam*)
Web of Science	TOPIC: (*fibromyalgia* OR *chronic, fatigue syndrome*) AND TOPIC: (*virtual reality* OR *exergame*)
SCOPUS	(TITLE-ABS-KEY (“fibromyalgia” OR “fibromyalgia syndrome” OR “chronic fatigue syndrome”) AND TITLE-ABS-KEY (“virtual reality” OR “exercises” OR “videogames”))
PEDro	Fibromyalgia AND virtual reality Fibromyalgia AND exergames
CINAHL Complete	AB (fibromyalgia OR fibromyalgia syndrome OR chronic fatigue syndrome) AND AB (virtual reality OR exergames OR videogames)

**Table 2:** Characteristics of the studies included in the review.

				Experimental Group								Control Group					Outcomes		
				Sample Characteristics				Intervention Characteristics				Sample Characteristics							
Authorship and Date	Country	K	N	N_e_	Age	BMI	Evol. Years	Type	Weeks	Ses/Week	Min	N_c_	Age	BMI	Evol. Years	Type Control	Variable	Test	Follow-Up
Collado-Mateo, D. et al., 2017a [62]	Spain	6	76	41	52.52	25.79	9.6	ni VRBT	8	2	60	35	52.47	27.75	11.02	NI	FMS Impact	FIQ	Inm. Effect
																	Quality of Life	EuroQol-5D	
																	Fatigue	FIQ-Fatigue	
																	Pain	FIQ-Pain	
																	Anx/Dep	VAS	
Collado-Mateo, D. et al., 2017b [64]	Spain	1	76	41	52.43	25.79	10.36	ni VRBT	8	2	60	35	52.58	27.75	12.48	NI	Dynamic Balance	TGUGT	Inm. Effect
García-Palacios, A. et al., 2015 [65]	Spain	4	59	30	50.48	NR	9.32	ni VRBT	3	2	60	29	50.48	NR	9.32	NI	FMS Impact	FIQ	Inm. Effect
																	Quality of Life	QLI-SP	
																	Pain	BPI	
																	Depression	BDI-II	
Gulsen, C. et al., 2020 [66]	Turkey	5	16	8	46.5	26.81	4	iVRBT + CTBTE	8	2	80	8	38.5	22.85	4	CTBTE	FMS Impact	FIQ	Inm. Effect
																	Pain	VAS	
																	Fatigue	FSS	
																	Aerobic capacity	6-MWT	
																	Quality of Life	SF-36	
León-Llamas, J.L. et al., 2020 [67]	Spain	1/ QS	50	25	54	27	8.5	ni VRBT	24	2	60	25	53	28.5	11	NI	Aerobic capacity	PVO_2_	Inm. Effect
Martín-Martínez, J.P. et al., 2019 [68]	Spain	1	55	28	54.04	27.36	19.2	ni VRBT	24	2	60	27	53.41	28.84	16.76	NI	Dynamic Balance	TGUGT	Inm. Effect
Polat, M. et al., 2021 [69]	Turkey	7	40	20	42.6	26.6	1.5	ni VRBT + CTBTE	8	3	35	20	47	27.9	1.4	CTBTE	FMS Impact	FIQ	Inm. Effect
																	Aerobic capacity	6-MWT	
																	Pain	VAS	
																	Fatigue	FSS	
																	Quality of Life	EQ-5D-5L	
																	Anx/Dep	HADS-A/-D	
Silva de Carvalho, M. et al., 2020 [61]	Brasil	4	21	11	55.64	30.28	9.91	ni VRBT	7	3	60	10	47.7	26.09	14.65	ST	FMS Impact	FIQ	Inm. Effect
																	Aerobic capacity	6-MWT	
																	Fatigue	FIQ-Fatigue	
																	Pain	FIQ-Pain	
Villafaina, S. et al., 2019a [63]	Spain	2	55	28	54.04	27.36	19.2	ni VRBT	24	2	60	27	53.41	28.84	16.74	NI	Pain	VAS	Inm. Effect
																	Quality of Life	EQ-5D-5L	
Villafaina, S. et al., 2019b [70]	Spain	3	37	22	54.27	27.1	NR	ni VRBT	24	2	60	15	53.44	28.19	NR	NI	FMS Impact	FIQ	Inm. Effect
																	Dynamic Balance	TGUGT	
																	Aerobic capacity	6-MWT	
Villafaina, S. et al., 2019c [71]	Spain	QS	50	25	52	NR	16	ni VRBT	24	2	60	25	54	NR	16	NI	Brain Dynamics	EEG Signals	Inm. Effect

Abbreviations: K, Number of comparisons provided; N, total sample size; Ne, experimental group sample size; BMI, body mass index; Evol, evolution; Ses, sSessions; Min, minutes; Nc, control group sample size; niVRBT, non-immersive virtual reality; iVR, immersive virtual reality; CTBTEM, conventional therapy based virtual training; ST, stretching exercise; FMS, fibromyalgia syndrome; FIQ, Fibromyalgia Impact Questionnaire; Anx, anxiety; Dep, depression; VAS, Visual Analogue Scale; TGUGT, Timed Get Up and Go Test; QLI-SP, Quality of Life Index; BPI, Brief Pain Inventory; BDI-II, Beck Depression Inventory II; FSS, Fatigue Severity Scale; EQ-5D-5L, The Euro Quality of Life Five Dimensions; HADS, Hospital Anxiety and Depression Scale-Anxiety Dimension; HADS-D, Hospital Anxiety and Depression Scale-Depression Dimension; EEG Signals, electroencephalographic signals.

**Table 3:** Methodological quality and risk of bias (PEDro Scores) of the studies included in the review.

Items	1	2	3	4	5	6	7	8	9	10	11	TOTAL
Authorship												
Collado-Mateo, D. et al., 2017a [62]	N	Y	N	Y	N	N	Y	Y	Y	Y	Y	7
Collado-Mateo, D. et al., 2017b [64]	Y	Y	Y	Y	N	N	Y	Y	N	Y	Y	7
García-Palacios, A. et al., 2015 [65]	Y	Y	N	Y	N	N	Y	Y	Y	Y	Y	7
Gulsen, C. et al., 2020 [66]	Y	Y	N	Y	N	N	N	Y	Y	Y	Y	6
León-Llamas, J.L. et al., 2020 [67]	Y	Y	N	Y	N	N	Y	Y	Y	Y	Y	7
Martín-Martínez, J.P. et al., 2019 [68]	Y	Y	N	Y	N	N	Y	Y	Y	Y	Y	7
Polat, M. et al., 2021 [69]	Y	Y	N	Y	N	N	Y	Y	Y	Y	Y	7
Silva de Carvalho, M. et al., 2020 [61]	Y	Y	N	Y	N	N	Y	N	Y	Y	Y	6
Villafaina, S. et al., 2019a [63]	Y	Y	N	Y	N	N	Y	Y	Y	Y	Y	7
Villafaina, S. et al., 2019b [70]	Y	Y	N	Y	N	N	Y	N	Y	Y	Y	6
Villafaina, S. et al., 2019c [71]	Y	Y	N	N	N	N	Y	Y	Y	Y	Y	6

Abbreviations: 1 = eligibility criteria, 2 = random allocation, 3 = concealed allocation, 4 = baseline comparability, 5 = blind subjects, 6 = blind therapists, 7 = blind assessors = 8, adequate follow-up, 9 = intention-to-treat analysis, 10 = between-group comparisons, 11 = point estimates and variability, Note = eligibility criteria item does not contribute to total score, Y = Yes, N = No.

**Table 4:** Main findings in meta-analyses.

Outcomes	Summary of Findings									Quality of Evidence (Grade)					
	Pooled Effect Het						Publication Bias								
	K	N	N_s_	SMD	95% CI	I^2^ (*p* for *Q*-test)	Funnel Plot (*p* for Egger)	Trim and Fill		Risk of Bias	Incons	Indirect	Imprec	Pub. Bias	Quality
								Adj SMD	% of Var						
Impact of FMS Symptoms	6	249	41.5	−0.62	−0.93 to −0.31	5.2% (*p* = 0.4)	Sym (*p* = 0.9)	−0.62	0%	Medium	No	No	Yes	No	Moderate
Pain	6	267	44.5	−0.45	−0.69 to −0.21	0% (*p* = 0.52)	Asym (*p* = 0.2)	−0.72	28%	Medium	No	No	Yes	Yes	Low
Dynamic Balance	3	168	56	−0.76	−1.12 to −0.39	4.4% (*p* = 0.35)	Sym (*p* = 0.52)	−0.75	0%	Medium	No	No	Yes	No	Low
Aerobic Capacity	5	164	32.8	0.32	0.004 to 0.63	0% (*p* = 0.57)	Asym (*p* = 0.31)	0.36	12%	Medium	No	No	Yes	Yes	Low
Fatigue	4	153	38.5	−0.58	−1.02 to −0.14	5.4% (*p* = 0.37)	Asym (*p* = 0.09)	−0.48	20%	Medium	No	No	Yes	Yes	Low
Quality of Life	5	246	49.2	0.55	0.3 to 0.81	0% (*p* = 0.73)	Sym (*p* = 0.9)	0.52	0%	Medium	No	No	Yes	No	Moderate
Anxiety	3	137	45.7	−0.47	−0.91 to −0.03	0% (*p* = 0.32)	Asym (*p* =0.2)	−0.57	22%	Medium	No	No	Yes	Yes	Very-Low
Depression	4	196	49	−0.46	−0.76 to −0.16	4.6% (*p* = 0.4)	Asym (*p* =0.14)	−0.52	13%	Medium	No	No	Yes	Yes	Low

Abbreviations: GRADE = grading of recommendations assessment, development, and evaluation, Het = heterogeneity, K = number of comparisons, N = total sample size, Ns = Participants per study, SMD = Cohen’s standardized mean difference, 95% CI = 95% confidence interval, I^2^ = degree of inconsistency *p* = *p*-value; Adj = adjusted, % of var = percentage of variation, Indirect = indirectness, Imprec = imprecision, Pub bias = publication bias, Sym = symmetric, Asym = asymmetric.

## References

[1] Wolfe F., Clauw D.J., Fitzcharles M.-A., Goldenberg D.L., Katz R.S., Mease P., Russell A.S., Russell I.J., Winfield J.B., Yunus M.B. (2010). The American College of Rheumatology Preliminary Diagnostic Criteria for Fibromyalgia and Measurement of Symptom Severity. Arthritis Rheum..

[2] Lomas-Vega R., Rodríguez-Almagro D., Peinado-Rubia A.B., Zagalaz-Anula N., Molina F., Obrero-Gaitán E., Ibáñez-Vera A.J., Osuna-Pérez M.C. (2020). Joint Assessment of Equilibrium and Neuromotor Function: A Validation Study in Patients with Fibromyalgia. Diagnostics.

[3] Montoro C.I., del Paso G.A.R., Duschek S. (2016). Alexithymia in fibromyalgia syndrome. Pers. Individ. Differ..

[4] Peinado-Rubia A., Osuna-Pérez M.C., Rodríguez-Almagro D., Zagalaz-Anula N., López-Ruiz M.C., Lomas-Vega R. (2020). Impaired Balance in Patients with Fibromyalgia Syndrome: Predictors of the Impact of This Disorder and Balance Confidence. Int. J. Environ. Res. Public Health.

[5] Sechi C., Lucarelli L., Vismara L. (2021). Depressive Symptoms and Quality of Life in a Sample of Italian Women with a Diagnosis of Fibromyalgia: The Role of Attachment Styles. Depress. Res. Treat..

[6] Kaleycheva N., Cullen A.E., Evans R., Harris T., Nicholson T., Chalder T. (2021). The role of lifetime stressors in adult fibromyalgia: Systematic review and meta-analysis of case-control studies. Psychol. Med..

[7] Gaudreault N., Boulay P. (2018). Cardiorespiratory fitness among adults with fibromyalgia. Breathe.

[8] Sechi C., Vismara L., Brennstuhl M.J., Tarquinio C., Lucarelli L. (2020). Adult attachment styles, self-esteem, and quality of life in women with fibromyalgia. Health Psychol. Open.

[9] Offenbaecher M., Kohls N., Ewert T., Sigl C., Hieblinger R., Toussaint L.L., Sirois F., Hirsch J., Vallejo M.A., Kramer S. (2021). Pain is not the major determinant of quality of life in fibromyalgia: Results from a retrospective “real world” data analysis of fibromyalgia patients. Rheumatol. Int..

[10] MacDougall P. (2021). In fibromyalgia, some therapies may provide small improvements in pain and quality of life. Ann. Intern. Med..

[11] Clauw D.J. (2014). Fibromyalgia: A clinical review. JAMA.

[12] Wolfe F., Walitt B., Perrot S., Rasker J.J., Häuser W. (2018). Fibromyalgia diagnosis and biased assessment: Sex, prevalence and bias. PLoS ONE.

[13] Queiroz L.P. (2013). Worldwide Epidemiology of Fibromyalgia. Curr. Pain Headache Rep..

[14] Okifuji A., Hare B.D. (2013). Management of Fibromyalgia Syndrome: Review of Evidence. Pain Ther..

[15] Cabral C.M.N., Miyamoto G.C., Franco K.F.M., Bosmans J.E. (2021). Economic evaluations of educational, physical, and psychological treatments for fibromyalgia. Pain.

[16] Casas-Barragán A., Molina F., Tapia-Haro R.M., García-Ríos M.C., Correa-Rodríguez M., Aguilar-Ferrándiz M.E. (2021). Association of core body temperature and peripheral blood flow of the hands with pain intensity, pressure pain hypersensitivity, central sensitization, and fibromyalgia symptoms. Ther. Adv. Chronic Dis..

[17] Rehm S., Sachau J., Hellriegel J., Forstenpointner J., Jacobsen H.B., Harten P., Gierthmühlen J., Baron R. (2021). Pain matters for central sensitization: Sensory and psychological parameters in patients with fibromyalgia syndrome. PAIN Rep..

[18] Pidal-Miranda M., González-Villar A.J., Carrillo-De-La-Peña M.T. (2019). Pain Expressions and Inhibitory Control in Patients with Fibromyalgia: Behavioral and Neural Correlates. Front. Behav. Neurosci..

[19] Russell I.J., Larson A.A. (2009). Neurophysiopathogenesis of Fibromyalgia Syndrome: A Unified Hypothesis. Rheum. Dis. Clin. North Am..

[20] Harte S.E., Harris R.E., Clauw D.J. (2018). The neurobiology of central sensitization. J. Appl. Biobehav. Res..

[21] Uygur-Kucukseymen E., Castelo-Branco L., Pacheco-Barrios K., Luna-Cuadros M.A., Cardenas A., Giannoni-Luza S., Zeng H., Gianlorenco A.C., Gnoatto-Medeiros M., Shaikh E.S. (2020). Decreased neural inhibitory state in fibromyalgia pain: A cross-sectional study. Neurophysiol. Clin. Neurophysiol..

[22] Littlejohn G., Guymer E. (2018). Neurogenic inflammation in fibromyalgia. Semin. Immunopathol..

[23] Chiu I., Von Hehn C.A., Woolf C.J. (2012). Neurogenic inflammation and the peripheral nervous system in host defense and immunopathology. Nat. Neurosci..

[24] Macfarlane G.J., Kronisch C., Dean L., Atzeni F., Häuser W., Flüß E., Choy E., Kosek E., Amris K., Branco J. (2016). EULAR revised recommendations for the management of fibromyalgia. Ann. Rheum. Dis..

[25] Del-Moral-García M., Obrero-Gaitán E., Rodríguez-Almagro D., Rodríguez-Huguet M., Osuna-Pérez M.C., Lomas-Vega R. (2020). Effectiveness of Active Therapy-Based Training to Improve the Balance in Patients with Fibromyalgia: A Systematic Review with Meta-Analysis. J. Clin. Med..

[26] Sosa-Reina M.D., Nunez-Nagy S., Gallego-Izquierdo T., Pecos-Martín D., Monserrat J., Álvarez-Mon M. (2017). Effectiveness of Therapeutic Exercise in Fibromyalgia Syndrome: A Systematic Review and Meta-Analysis of Randomized Clinical Trials. BioMed. Res. Int..

[27] Berardi G., Senefeld J.W., Hunter S.K., Bement M.K.H. (2021). Impact of isometric and concentric resistance exercise on pain and fatigue in fibromyalgia. Graefe’s Arch. Clin. Exp. Ophthalmol..

[28] Izquierdo-Alventosa R., Inglés M., Cortés-Amador S., Gimeno-Mallench L., Chirivella-Garrido J., Kropotov J., Serra-Añó P. (2020). Low-Intensity Physical Exercise Improves Pain Catastrophizing and Other Psychological and Physical Aspects in Women with Fibromyalgia: A Randomized Controlled Trial. Int. J. Environ. Res. Public Health.

[29] Caglayan B.C., Keskin A., Kabul E.G., Calik B.B., Aslan U.B., Karasu U. (2021). Effects of clinical Pilates exercises in individuals with fibromyalgia: A randomized controlled trial. Eur. J. Rheumatol..

[30] Costa M.T.S., Vieira L.P., Barbosa E.D.O., Oliveira L.M., Maillot P., Vaghetti C.A.O., Carta M.G., Machado S., Gatica-Rojas V., Monteiro-Junior R.S. (2019). Virtual Reality-Based Exercise with Exergames as Medicine in Different Contexts: A Short Review. Clin. Pract. Epidemiol. Ment. Health.

[31] Corbetta D., Imeri F., Gatti R. (2015). Rehabilitation that incorporates virtual reality is more effective than standard rehabilitation for improving walking speed, balance and mobility after stroke: A systematic review. J. Physiother..

[32] Palacios-Navarro G., Hogan N. (2021). Head-Mounted Display-Based Therapies for Adults Post-Stroke: A Systematic Review and Meta-Analysis. Sensors.

[33] Montoro-Cárdenas D., Cortés-Pérez I., Zagalaz-Anula N., Osuna-Pérez M.C., Obrero-Gaitán E., Lomas-Vega R. (2021). Nintendo Wii Balance Board therapy for postural control in children with cerebral palsy: A systematic review and meta-analysis. Dev. Med. Child Neurol..

[34] Cortés-Pérez I., Zagalaz-Anula N., Montoro-Cárdenas D., Lomas-Vega R., Obrero-Gaitán E., Osuna-Pérez M. (2021). Leap Motion Controller Video Game-Based Therapy for Upper Extremity Motor Recovery in Patients with Central Nervous System Diseases. A Systematic Review with Meta-Analysis. Sensors.

[35] Zhang B., Li D., Liu Y., Wang J., Xiao Q. (2021). Virtual reality for limb motor function, balance, gait, cognition and daily function of stroke patients: A systematic review and meta-analysis. J. Adv. Nurs..

[36] Ahern M.M., Dean L.V., Stoddard C.C., Agrawal A., Kim K., Cook C.E., Garcia A.N. (2020). The Effectiveness of Virtual Reality in Patients with Spinal Pain: A Systematic Review and Meta-Analysis. Pain Pract..

[37] Hwang R., Elkins M.R. (2020). Telephysiotherapy. J. Physiother..

[38] Darnall B.D., Krishnamurthy P., Tsuei J., Minor J.D. (2020). Self-Administered Skills-Based Virtual Reality Intervention for Chronic Pain: Randomized Controlled Pilot Study. JMIR Form. Res..

[39] Herrero R., García-Palacios A., Castilla D., Molinari G., Botella C. (2014). Virtual Reality for the Induction of Positive Emotions in the Treatment of Fibromyalgia: A Pilot Study over Acceptability, Satisfaction, and the Effect of Virtual Reality on Mood. Cyberpsychology Behav. Soc. Netw..

[40] Mortensen J., Kristensen L.Q., Brooks E.P., Brooks A.L. (2013). Women with fibromyalgia’s experience with three motion-controlled video game consoles and indicators of symptom severity and performance of activities of daily living. Disabil. Rehabil. Assist. Technol..

[41] Moher D., Liberati A., Tetzlaff J., Altman D.G. (2009). Preferred Reporting Items for Systematic Reviews and Meta-Analyses: The PRISMA Statement. J. Clin. Epidemiol..

[42] Higgins J.P.T., Green S. (2011). Cochrane Handbook for Systematic Reviews of Intervention Version 5.1.0.

[43] Maher C.G., Sherrington C., Herbert R.D., Moseley A.M., Elkins M. (2003). Reliability of the PEDro Scale for Rating Quality of Randomized Controlled Trials. Phys. Ther..

[44] Macedo L.G., Elkins M., Maher C., Moseley A.M., Herbert R., Sherrington C. (2010). There was evidence of convergent and construct validity of Physiotherapy Evidence Database quality scale for physiotherapy trials. J. Clin. Epidemiol..

[45] Elkins M.R., Moseley A.M., Sherrington C., Herbert R.D., Maher C.G. (2012). Growth in the Physiotherapy Evidence Database (PEDro) and use of the PEDro scale. Br. J. Sports Med..

[46] Atkins D., Best D., Briss P., Eccles M., Falck-Ytter Y., Flottorp S., Guyatt G., Harbour R., Haugh M., Henry D. (2004). Grading quality of evidence and strength of recommendations. BMJ.

[47] Meader N., King K., Llewellyn A., Norman G., Brown J., Rodgers M., Moe-Byrne T., Higgins J.P., Sowden A., Stewart G. (2014). A checklist designed to aid consistency and reproducibility of GRADE assessments: Development and pilot validation. Syst. Rev..

[48] Higgins J.P.T., Thompson S.G., Deeks J., Altman D.G. (2003). Measuring inconsistency in meta-analyses. BMJ.

[49] Borenstein M., Hedges L., Higgins J., Rothstein H. (2020). Comprehensive Meta-Analysis Software Version 3.

[50] Cooper H., Hedges L.V., Valentine J.C. (2009). The Handbook of Research Synthesis and Meta-Analysis.

[51] Cohen J. (1977). Statistical Power Analysis for the Behavioral Sciences.

[52] Der Simonian R., Laird N. (1986). Meta-analysis in clinical trials. Control. Clin. Trials.

[53] Faraone S.V. (2008). Interpreting estimates of treatment effects: Implications for managed care. Pharm. Ther..

[54] Rücker G., Schwarzer G. (2020). Beyond the forest plot: The drapery plot. Res. Synth. Methods.

[55] Sterne J.A., Egger M. (2001). Funnel plots for detecting bias in meta-analysis: Guidelines on choice of axis. J. Clin. Epidemiol..

[56] Egger M., Smith G.D., Schneider M., Minder C. (1997). Bias in meta-analysis detected by a simple, graphical test. BMJ.

[57] Duval S., Tweedie R. (2000). Trim and Fill: A Simple Funnel-Plot-Based Method of Testing and Adjusting for Publication Bias in Meta-Analysis. Biometrics.

[58] Rothman K.J., Greenland S., Lash T.L. (2008). Modern Epidemiology.

[59] Higgins J., Thompson S., Deeks J., Altman D. (2002). Statistical heterogeneity in systematic reviews of clinical trials: A critical appraisal of guidelines and practice. J. Health Serv. Res. Policy.

[60] Siddaway A.P., Wood A.M., Hedges L.V. (2019). How to Do a Systematic Review: A Best Practice Guide for Conducting and Reporting Narrative Reviews, Meta-Analyses, and Meta-Syntheses. Annu. Rev. Psychol..

[61] de Carvalho M.S., Carvalho L.C., Menezes F.D.S., Frazin A., Gomes E.D.C., Iunes D.H. (2020). Effects of Exergames in Women with Fibromyalgia: A Randomized Controlled Study. Games Health J..

[62] Collado-Mateo D., Dominguez-Muñoz F.J., Adsuar J.C., Garcia-Gordillo M.A., Gusi N. (2017). Effects of Exergames on Quality of Life, Pain, and Disease Effect in Women with Fibromyalgia: A Randomized Controlled Trial. Arch. Phys. Med. Rehabil..

[63] Villafaina S., Collado-Mateo D., Fuentes J.P., Rohlfs-Domínguez P., Gusi N. (2019). Effects of Exergames on Brain Dynamics in Women with Fibromyalgia: A Randomized Controlled Trial. J. Clin. Med..

[64] Collado-Mateo D., Dominguez-Muñoz F.J., Adsuar J.C., Merellano-Navarro E., Gusi N. (2017). Exergames for women with fibromyalgia: A randomised controlled trial to evaluate the effects on mobility skills, balance and fear of falling. PeerJ.

[65] Garcia-Palacios A., Herrero R., Vizcaíno Y., Belmonte M.A., Castilla D., Molinari G., Baños R.M., Botella C. (2015). Integrating Virtual Reality with Activity Management for the Treatment of Fibromyalgia. Clin. J. Pain.

[66] Gulsen C., Soke F., Eldemir K., Apaydin Y., Ozkul C., Guclu-Gunduz A., Akcali D.T. (2020). Effect of fully immersive virtual reality treatment combined with exercise in fibromyalgia patients: A randomized controlled trial. Assist. Technol..

[67] Leon-Llamas J., Villafaina S., Murillo-Garcia A., Dominguez-Muñoz F., Gusi N. (2020). Effects of 24-Week Exergame Intervention on the Gray Matter Volume of Different Brain Structures in Women with Fibromyalgia: A Single-Blind, Randomized Controlled Trial. J. Clin. Med..

[68] Martín-Martínez J.P., Villafaina S., Collado-Mateo D., Pérez-Gómez J., Gusi N. (2019). Effects of 24-week exergame intervention on physical function under single- and dual-task conditions in fibromyalgia: A randomized controlled trial. Scand. J. Med. Sci. Sports.

[69] Polat M., Kahveci A., Muci B., Günendi Z., Karataş G.K. (2021). The Effect of Virtual Reality Exercises on Pain, Functionality, Cardiopulmonary Capacity, and Quality of Life in Fibromyalgia Syndrome: A Randomized Controlled Study. Games Health J..

[70] Villafaina S., Collado-Mateo D., Domínguez-Muñoz F.J., Fuentes-García J.P., Gusi N. (2019). Benefits of 24-Week Exergame Intervention on Health-Related Quality of Life and Pain in Women with Fibromyalgia: A Single-Blind, Randomized Controlled Trial. Games Health J..

[71] Villafaina S., Borrega-Mouquinho Y., Fuentes-García J.P., Collado-Mateo D., Gusi N. (2019). Effect of Exergame Training and Detraining on Lower-Body Strength, Agility, and Cardiorespiratory Fitness in Women with Fibromyalgia: Single-Blinded Randomized Controlled Trial. Int. J. Environ. Res. Public Health.

[72] Cipolletta S., Tomaino S.C.M., Magno E.L., Faccio E. (2020). Illness Experiences and Attitudes towards Medication in Online Communities for People with Fibromyalgia. Int. J. Environ. Res. Public Health.

[73] Cao C.-F., Ma K.-L., Li Q.-L., Luan F.-J., Wang Q.-B., Zhang M.-H., Viswanath O., Myrcik D., Varrassi G., Wang H.-Q. (2021). Balneotherapy for Fibromyalgia Syndrome: A Systematic Review and Meta-Analysis. J. Clin. Med..

[74] Masquelier E., D’Haeyere J. (2021). Physical activity in the treatment of fibromyalgia. Jt. Bone Spine.

[75] Busch A.J., Webber S., Richards R.S., Bidonde J., Schachter C.L., Schafer L., Danyliw A., Sawant A., Bello-Haas V.D., Rader T. (2013). Resistance exercise training for fibromyalgia. Cochrane Database Syst. Rev..

[76] Bennett R.M., Friend R., Jones K.D., Ward R., Han B.K., Ross R.L. (2009). The Revised Fibromyalgia Impact Questionnaire (FIQR): Validation and psychometric properties. Arthritis Res. Ther..

[77] Gumaa M., Youssef A.R. (2019). Is Virtual Reality Effective in Orthopedic Rehabilitation? A Systematic Review and Meta-Analysis. Phys. Ther..

[78] Ahmadpour N., Randall H., Choksi H., Gao A., Vaughan C., Poronnik P. (2019). Virtual Reality interventions for acute and chronic pain management. Int. J. Biochem. Cell Biol..

[79] Dunn J., Yeo E., Moghaddampour P., Chau B., Humbert S. (2017). Virtual and augmented reality in the treatment of phantom limb pain: A literature review. NeuroRehabilitation.

[80] Zusman M. (2002). Forebrain-mediated sensitization of central pain pathways: ‘non-specific’ pain and a new image for MT. Man. Ther..

[81] Goldman-Rakic P.S. (1996). The prefrontal landscape: Implications of functional architecture for understanding human mentation and the central executive. Philos. Trans. R. Soc. B Biol. Sci..

[82] Koçyiğit B.F., Akaltun M.S. (2020). Kinesiophobia Levels in Fibromyalgia Syndrome and the Relationship Between Pain, Disease Activity, Depression. Arch. Rheumatol..

[83] Núñez-Fuentes D., Obrero-Gaitán E., Zagalaz-Anula N., Ibáñez-Vera A., Achalandabaso-Ochoa A., López-Ruiz M., Rodríguez-Almagro D., Lomas-Vega R. (2021). Alteration of Postural Balance in Patients with Fibromyalgia Syndrome—A Systematic Review and Meta-Analysis. Diagnostics.

[84] Cagnie B., Coppieters I., Denecker S., Six J., Danneels L., Meeus M. (2014). Central sensitization in fibromyalgia? A systematic review on structural and functional brain MRI. Semin. Arthritis Rheum..

[85] Cheng C.-A., Chiu Y.-W., Wu D., Kuan Y.-C., Chen S.-N., Tam K.-W. (2019). Effectiveness of Tai Chi on fibromyalgia patients: A meta-analysis of randomized controlled trials. Complement. Ther. Med..

[86] Walitt B., Klose P., Üçeyler N., Phillips T., Häuser W. (2016). Antipsychotics for fibromyalgia in adults. Cochrane Database Syst. Rev..

[87] Dailey D.L., Law L.A.F., Vance C.G.T., Rakel B.A., Merriwether E.N., Darghosian L., Golchha M., Geasland K.M., Spitz R., Crofford L.J. (2016). Perceived function and physical performance are associated with pain and fatigue in women with fibromyalgia. Arthritis Res..

[88] Ioannou A., Papastavrou E., Avraamides M.N., Charalambous A. (2020). Virtual Reality and Symptoms Management of Anxiety, Depression, Fatigue, and Pain: A Systematic Review. SAGE Open Nurs..

[89] Vance C.G., Zimmerman M.B., Dailey D.L., Rakel B.A., Geasland K.M., Chimenti R.L., Williams J.M., Golchha M., Crofford L.J., Sluka K.A. (2020). Reduction in movement-evoked pain and fatigue during initial 30-minute transcutaneous electrical nerve stimulation treatment predicts transcutaneous electrical nerve stimulation responders in women with fibromyalgia. Pain.

[90] Montana J.I., Matamala-Gomez M., Maisto M., Mavrodiev P.A., Cavalera C.M., Diana B., Mantovani F., Realdon O. (2020). The Benefits of emotion Regulation Interventions in Virtual Reality for the Improvement of Wellbeing in Adults and Older Adults: A Systematic Review. J. Clin. Med..

[91] Arcos-Carmona I.M., Castro-Sánchez A.M., Matarán-Peñarrocha G.A., Gutiérrez-Rubio A.B., Ramos-González E., Moreno-Lorenzo C. (2011). Effects of aerobic exercise program and relaxation techniques on anxiety, quality of sleep, depression, and quality of life in patients with fibromyalgia: A randomized controlled trial. Med. Clin..

[92] Van Abbema R., Van Wilgen C.P., Van Der Schans C.P., Van Ittersum M.W. (2010). Patients with more severe symptoms benefit the most from an intensive multimodal programme in patients with fibromyalgia. Disabil. Rehabil..

[93] Freeman D., Reeve S., Robinson A., Ehlers A., Clark D., Spanlang B., Slater M. (2017). Virtual reality in the assessment, understanding, and treatment of mental health disorders. Psychol. Med..

[94] Fodor L.A., Coteț C.D., Cuijpers P., Szamoskozi T., David D., Cristea I.A. (2018). The effectiveness of virtual reality based interventions for symptoms of anxiety and depression: A meta-analysis. Sci. Rep..

[95] Masaoka Y., Homma I. (2000). The source generator of respiratory-related anxiety potential in the human brain. Neurosci. Lett..

[96] Gormsen L., Rosenberg R., Bach F., Jensen T.S. (2010). Depression, anxiety, health-related quality of life and pain in patients with chronic fibromyalgia and neuropathic pain. Eur. J. Pain.

[97] Thieme K., Turk D.C., Flor H. (2004). Comorbid Depression and Anxiety in Fibromyalgia Syndrome: Relationship to Somatic and Psychosocial Variables. Psychosom. Med..

[98] Montoro C.I., Duschek S., Schuepbach D., Gandarillas M., del Paso G.A.R. (2018). Cerebral blood flow variability in fibromyalgia syndrome: Relationships with emotional, clinical and functional variables. PLoS ONE.

[99] Rodriguez A., Tembl J., Mesa-Gresa P., Muñoz M., Montoya P., Rey B. (2017). Altered cerebral blood flow velocity features in fibromyalgia patients in resting-state conditions. PLoS ONE.

[100] Galvez-Sánchez C.M., del Paso G.A.R., Duschek S. (2018). Cognitive Impairments in Fibromyalgia Syndrome: Associations with Positive and Negative Affect, Alexithymia, Pain Catastrophizing and Self-Esteem. Front. Psychol..

[101] Van Liew C., Leon G., Neese M., Cronan T.A. (2018). You get used to it, or do you: Symptom length predicts less fibromyalgia physical impairment, but only for those with above-average self-efficacy. Psychol. Health Med..

